# Prioritizing symptom management in the treatment of chronic heart failure

**DOI:** 10.1002/ehf2.12875

**Published:** 2020-08-05

**Authors:** Aaron O. Koshy, Elisha R. Gallivan, Melanie McGinlay, Sam Straw, Michael Drozd, Anet G. Toms, John Gierula, Richard M. Cubbon, Mark T. Kearney, Klaus K. Witte

**Affiliations:** ^1^ Leeds Institute of Cardiovascular and Metabolic Medicine University of Leeds Clarendon Way Leeds LS2 9NL UK; ^2^ Department of Cardiology Leeds Teaching Hospitals NHS Trust Leeds UK

**Keywords:** Chronic heart failure, Symptom assessment, Quality of life, Patient‐reported outcomes

## Abstract

Chronic heart failure (CHF) is a chronic, progressive disease that has detrimental consequences on a patient's quality of life (QoL). In part due to requirements for market access and licensing, the assessment of current and future treatments focuses on reducing mortality and hospitalizations. Few drugs are available principally for their symptomatic effect despite the fact that most patients' symptoms persist or worsen over time and an acceptance that the survival gains of modern therapies are mitigated by poorly controlled symptoms. Additional contributors to the failure to focus on symptoms could be the result of under‐reporting of symptoms by patients and carers and a reliance on insensitive symptomatic categories in which patients frequently remain despite additional therapies. Hence, formal symptom assessment tools, such as questionnaires, can be useful prompts to encourage more fidelity and reproducibility in the assessment of symptoms. This scoping review explores for the first time the assessment options and management of common symptoms in CHF with a focus on patient‐reported outcome tools. The integration of patient‐reported outcomes for symptom assessment into the routine of a CHF clinic could improve the monitoring of disease progression and QoL, especially following changes in treatment or intervention with a targeted symptom approach expected to improve QoL and patient outcomes.

## Introduction

**Figure 1:**
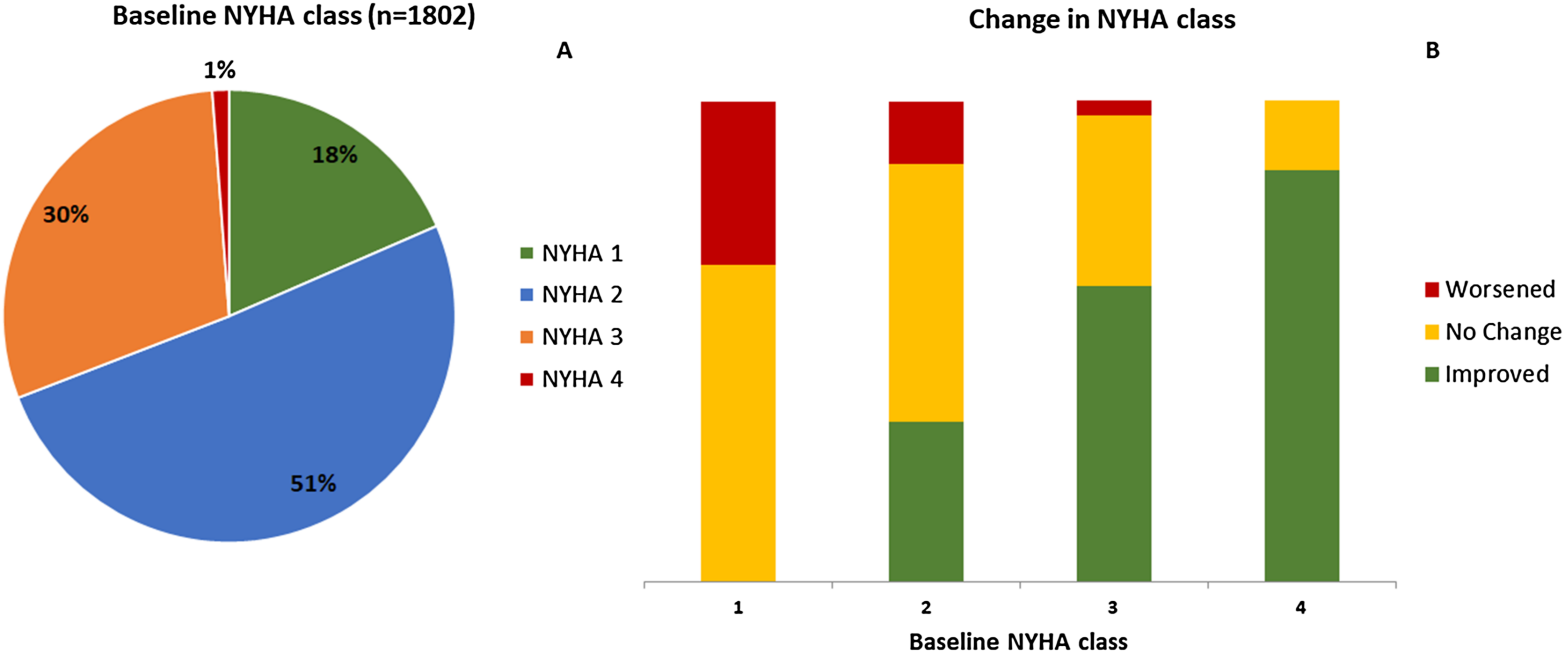
The distribution of heart failure patients by New York Heart Association (NYHA) at (A) baseline visit and (B) change after 1 year of follow‐up at a specialist heart failure clinic.

Chronic heart failure (CHF) is a progressive, debilitating disease characterized by persistently reduced exercise capacity and acute exacerbations that lead to repeated hospital admissions.^1^ More than 26 million people are estimated to be living with CHF worldwide, with a prevalence of ~1–2% in Europe.^2^ A globally ageing population is likely to increase these figures, increasing financial and resource pressures within healthcare systems.^2^, ^3^ Guideline‐approved treatments mostly focus on reducing mortality and hospitalization and preventing progressive adverse cardiac remodelling.^4^, ^5^ Despite optimal medical management and device therapy, patients often have persistent symptoms and long‐term reductions in quality of life (QoL)^5^, ^6^, ^7^ as evidenced in 400 CHF patients with serial assessments from our own published data (*Figure* *1*)^8^ in which a significant portion continues to have symptoms despite optimal therapy, and over 50% of those in New York Heart Association (NYHA) Class II patients do not improve. Allen *et al*.^9^ obtained similar findings using the American PINNACLE Registry in which the trend was towards worsening symptoms rather than a reduction over 2 years of follow‐up.While dyspnoea, fatigue, and oedema are classed as hallmark symptoms, pain, low mood, and chronic cough are also commonly reported by patients.^10^ These symptoms significantly impose on QoL and energy levels^11^ and are generally the reason for referral to specialist care. The symptom burden for CHF patients has been likened to those with advanced cancer or acquired immune deficiency syndrome,^6^, ^12^ yet in the months prior to death, cancer patients receive more frequent palliative care consultations and symptom‐directed prescriptions and report a lower impact of symptoms than those with CHF.^13^ Even mild symptoms of CHF can directly worsen patients' ability to manage daily activities including self‐care and adherence to recommended treatment.^14^ As symptoms worsen, many CHF patients become dependent on carers, which adversely affects their sense of identity and will to live.^6^, ^15^The Evaluation Study of Congestive Heart Failure and Pulmonary Artery Catheterization Effectiveness (ESCAPE) trial^16^ interviewed 287 patients from initial hospitalization until 6 months of post‐admission revealing that shortly after discharge, more than half were willing to trade survival time for improved symptom control, but once their symptoms had stabilized beyond 6 months, the majority (68%) prioritized survival. Factors associated with willingness to trade time included symptom severity and a higher depression score within the Minnesota Living with Heart Failure Questionnaire (MLHFQ). These data are not the only to suggest that as CHF progresses, patients are increasingly willing to trade time for symptom control.^17^Symptom control is also of economic relevance. The cost of care of patients with CHF is overwhelmingly due to hospitalization,^18^, ^19^ and this is largely for symptom control. Prioritizing symptom management in clinics could reduce hospital readmissions and reduce the costs of care.^1^, ^20^ There are in fact many treatments proven to improve symptoms, but they are infrequently employed because of a neutral (or negative) effect on disease progression including diuretics, dobutamine, and morphine.^21^We propose that a shift in focus in clinical care and research towards symptom assessment and targeted management could improve QoL and quality of life years while also being highly cost‐effective and that new treatments should be assessed and considered for approval based upon their effect on symptoms rather than simply survival. However, such a shift in priority will depend upon reliable, sensitive, and reproducible assessments of both classical and atypical symptoms of CHF. Hence, in this article, we discuss the common and less common symptoms of CHF and review the tools currently available for their assessment with the aim of prompting a greater focus on symptoms.

## Methods

For this scoping review, we undertook a protocolized PubMed search to identify articles published from 1946 to October 2019 including the following search terms: Heart failure, CHF, symptoms, relief, treatment, management, quality of life, QoL, oedema, edema, swelling, fluid, fatigue, weak, cough, dyspnoea, short of breath, breathlessness, SoB, depression, mood, exercise intolerance, exercise capacity, exercise testing, patient reported outcomes, and PRO. Grey literature was also searched using Google Search and Google Scholar. The abstracts of these articles were reviewed and considered for inclusion by the two first authors (AOK and ERG) based upon the relevance of the symptom, the description, practicality of the assessment tool, method of assessment, and the interventions for which it had been applied.This article summarizes our findings: first discussing each of the common complaints found in CHF with basic pathophysiology and then examples of how these can be assessed. *Table* *1* provides an overview of the validated symptom assessment tools in the context of CHF. We considered a tool as validated if proven reliable against the gold standard or if there is a published test–retest reliability coefficient (or Cronbach's alpha) greater than 0.7. Finally, where we discuss QoL, this is in the context of health and specifically to heart failure unless stated otherwise.

**Table 1:** Summary of questionnaires validated for CHF that focus on the common complaints covered in this review

Symptom assessed	Name of assessment tool with validation reference	Description	Structure	Strengths	Limitations
Fatigue	Dutch Exertion Fatigue Scale^22^	Assesses exertional fatigue	● 9‐item questionnaire with participants grading across 5 responses from 0 (no) to 4 (yes)	● Simple to use	● Limited utilization outside of Dutch speaking counties
				● Able to assess exertional fatigue	
				● Translated in four languages	
	Dutch Fatigue Scale^22^	Assesses general fatigue	● 9‐item questionnaire with responses graded from 1 to 5 on a Likert scale. This is aggregated to produce a total score ranging from 9 to 45, indicating increased fatigue.	● Simple to use	● Limited utilization outside of Dutch speaking counties despite translation available in four languages
Fatigue and Dyspnoea	Dyspnoea–Fatigue Index^a^ ^23^	Assessed the magnitude of fatigue or dyspnoea	● Three component questions scored from 0 to 4 based on the magnitude of the task that produces fatigue or dyspnoea. The score is aggregated from 0 (worst) to 12 (best).	● Simple to use	● Should not be used if other physical or cognitive factors can affect task, effort, or function
Dyspnoea	BDI and TDI^b^ ^24^	Assesses dyspnoea in relation to ADL	● BDI is developed from cumulative scores given by patients who assign a grade of 0–4 (0 = significant impairment; 4 = no impairment) for various tasks.	● Determines what degree of activity provokes dyspnoea	● Questions are not standardized, making the instrument user dependent resulting in potential interviewer bias.
			● Used in tandem with TDI to track changes in dyspnoea		
	Dyspnoea‐12^25^	Assesses the patient's perceptions and extent of dyspnoea experienced	● Dyspnoea is rated ‘none’ to ‘severe’ across 12 potential associations of the symptom such as a sensation of exhaustion or distress.	● Easy to use	● The tool is not recommended if more than three questions are left unanswered.
				● Patient specific	
				● Assesses multiple components of dyspnoea	● Unclear link between psychological distress and perceived breathlessness severity
	New York Heart Association functional classification^c^ ^26^	Assesses limitations in physical activity manifesting as dyspnoea	● Graded from 1 (no dyspnoea at strenuous exertion) to 4 (symptoms at rest)	● Easy to use	● Inter‐operator variability
				● Internationally recognized	
				● Associated with prognosis	
				● Validated extended versions	
Low mood	Beck Depression Inventory^27^	Assesses patient for depressive symptoms	● 21‐item assessment scoring depressive symptoms from 0 to 3	● Well validated across cardiac patients	● Lengthy
	Cardiac Depression Scale^28^	Assesses depressed mood in cardiac patients	● 26‐item assessment requiring a response from 1 (strongly disagree) to 7 (strongly agree)	● Well validated across cardiac patients	● Limited utilization in CHF research
	Geriatric Depression Scale–Short form^28^	Assesses patient for depressive symptoms	● 15‐item self‐assessment scale consisting of yes/no questions	● Well validated across age groups and languages as repeatable and responsive	● Potential variance in different ethnic groups
			● Overall maximum score of 15, with 5 and above indicating a diagnosis of depressive disorder	● Concise and self‐administered	
	Hospital Anxiety and Depression Scale^29^	Assesses patient depressive and anxiety symptoms	● 14‐item self‐assessment scale	● Concise and self‐administered, therefore practical for clinical use	● Limited validation in large Danish cohort
	Patient Health Questionnaire‐9^30^	Assesses patient for depressive symptoms	● 10‐item self‐reporting questionnaire	● Well validated, repeatable, and responsive	● Unclear cut‐off rate for screening and accuracy
				● Concise and self‐administered	
			● Patients answer questions using a score from 0 (not at all) to 3 (nearly every day).	● Correlates with readmission and QoL	

ADL, activities of daily living; BDI, Baseline Dyspnoea Index; CHF, chronic heart failure; QoL, quality of life; TDI, Transition Dyspnoea Index.

^a^ Also known as the Index of Dyspnoea–Fatigue (IDF) of Yale University or Feinstein's Index of Dyspnoea with other versions known as Yale Dyspnoea–Fatigue Index and Yale Scale.

^b^ Validated in patients with CHF and gastrointestinal symptoms.

^c^ Validated historically and more recently as an extended form of seven questions.

## Exercise intolerance

**Figure 2:**
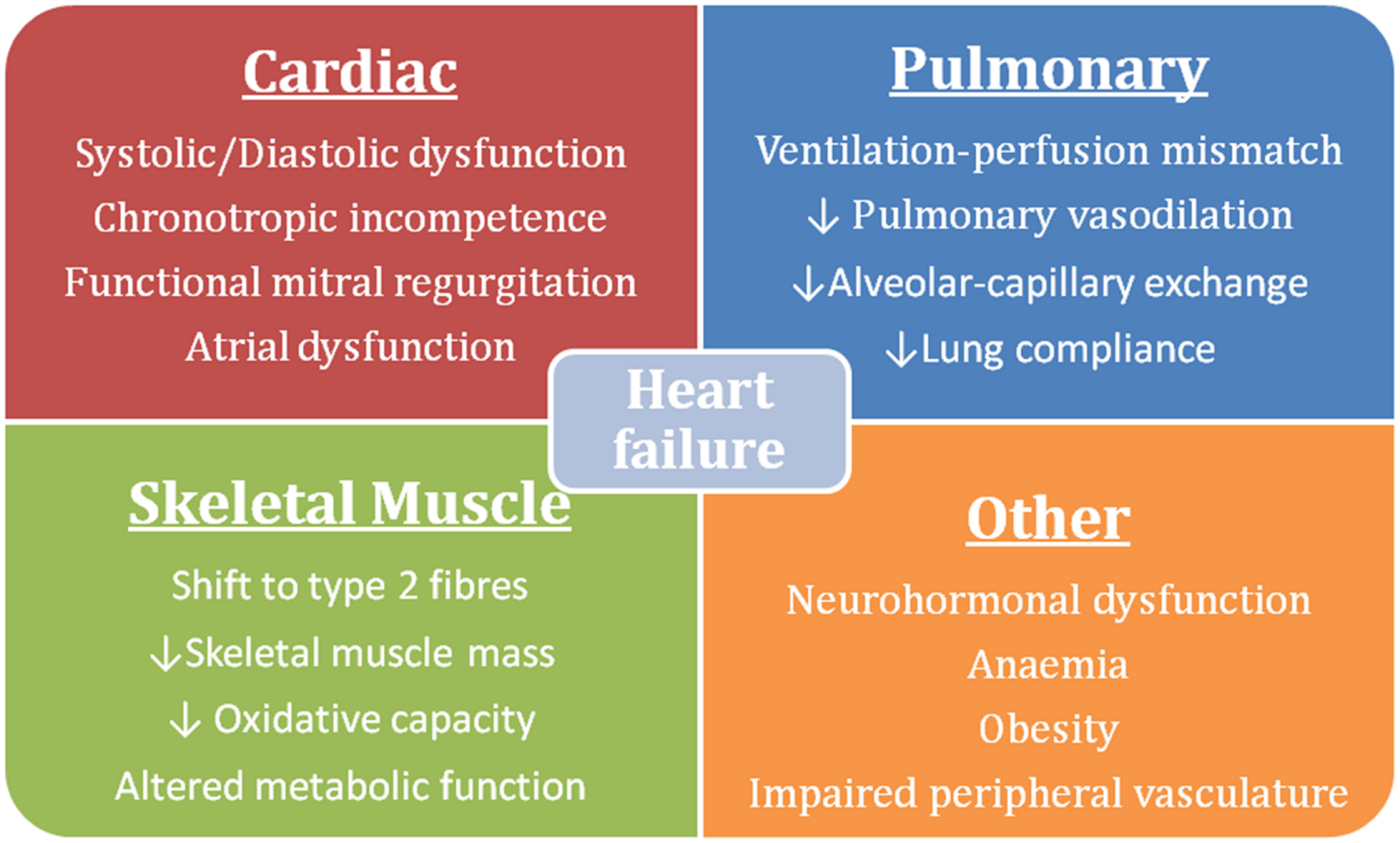
Common contributors to reduced exercise capacity in patients with chronic heart failure.

Exercise intolerance is the inability to conduct physical exertion at a ‘normal’ level and is by far the most common symptom of CHF.^7^ The degree of reduction in exercise capacity relates to both worse prognosis and QoL.^31^ Exercise intolerance is due to a combination of central factors such as heart rate and stroke volume as well as peripheral factors including skeletal muscle structure and function (*Figure* *2*),^32^, ^33^, ^34^ manifesting as fatigue or dyspnoea.Exercise capacity can be determined relatively consistently in clinics using semi‐quantitative and objective methods including the NYHA functional classification, the 6 min walk test, and cardiopulmonary exercise testing.^35^ Measures of exercise capacity outperform echocardiography in prognostic assessment.^36^ Furthermore, relatively small improvements in exercise time are associated with a lower hospitalization rate, superior QoL, and improved survival in the long term.^37^ The low sensitivity of NYHA classification and 6 min walk test limits their ability to measure change over time, while the additional equipment to measure metabolic gas exchange limits the widespread applicability of cardiopulmonary exercise testing.^38^, ^39^ We prefer a simpler measure of direct patient relevance—exercise time on a treadmill or cycle—which has high reproducibility and can easily be converted to distance.^40^Exercise capacity is a common endpoint for interventions in CHF. A number of treatments ranging from pharmacological such as intravenous iron, device therapies such as cardiac resynchronization therapy, and non‐pharmacological options such aerobic exercise are associated with improved exercise capacity.^41^, ^42^, ^43^ Standardized and simplified assessment of exercise capacity could allow for a more nuanced approach to the management of symptoms, enabling patients to have greater control of functional capacity and mortality.

## Fatigue

Fatigue is a hallmark symptom of CHF that affects ~85% of CHF patients.^10^ The origin is likely to stem from both skeletal muscles and central nervous system.It has been suggested, for example, that decreased cardiac output especially during activity leads to a greater oxygen or metabolic debt that lengthens recovery time possibly even to beyond the next activity.^44^ However, fatigue could also be due to sleep disturbance due to anxiety, pain, orthopnoea, or paroxysmal nocturnal dyspnoea.^1^, ^10^, ^45^Fatigue is difficult to treat.^7^, ^46^ With CHF predominantly affecting the elderly, fatigue is often dismissed as a consequence of ageing and deconditioning and is therefore poorly recognized or explored.^14^ Education of patients and carers on how to assess and manage fatigue could therefore improve patient‐orientated outcomes. Despite not being validated in CHF, the 20‐item Multidimensional Fatigue Inventory has been frequently utilized in CHF studies.^14^, ^47^ The tool is comprehensive and assesses general fatigue, physical fatigue, mental fatigue, reduced motivation, and reduced activity.^48^ However, a number of tools validated for CHF include fatigue as one of their domains (*Table* *1*).Fatigue has significant overlap with other symptoms found in CHF. As well as affecting functional status, fatigue is closely associated with depression; Falk *et al*.^14^ found that reduced activity was associated with low motivation in CHF patients. Significant dyspnoea also appears to worsen physical fatigue suggesting better symptom management could improve exercise intolerance and consequently mood.^14^There is limited guidance on how to effectively treat fatigue because emotional and psychological factors play a major role in the experience of physiological fatigue.^46^, ^49^ A multidisciplinary approach involving cardiologists, psychological support, physiotherapists, sleep specialists, and dieticians has been proposed.^10^

## Dyspnoea

Breathlessness is a key symptom of CHF. Goebel *et al*.^50^ found that 61.7% of 96 CHF patients reported shortness of breath with breathlessness being the most common complaint prompting a hospital consultation.^1^, ^10^, ^51^Dyspnoea is thought to be due to a combination of factors including diaphragmatic or skeletal muscle weakness, deconditioning, obesity, anaemia, pulmonary oedema, or lung stiffness due to elevated left ventricular pressure.^10^, ^45^, ^50^ Similar to fatigue, dyspnoea is often underappreciated and seen as a by‐product of ageing and reduced fitness.^45^ The patient experience of dyspnoea is highly variable and can significantly reduce morale.^10^ Breathlessness can be episodic or continuous, ranging from an uncomfortable awareness of breathing to a feeling of suffocation or breathlessness.^1^, ^10^, ^51^ It is often frightening and has a psychological impact.^10^, ^45^, ^52^, ^53^ A multivariable analysis of the COMET study found breathlessness to be the only symptom that was a significant predictor of mortality.^52^ Thus, the accurate assessment of dyspnoea is especially important due to its long‐term implications to hospitalization and prognosis.Dyspnoea is often ranked numerically or with ‘Likert’ scales based on how it impacts activities of daily living and thereby QoL.^10^, ^51^ The commonly utilized NYHA class has variable sensitivity in gauging dyspnoea and classifying patients.^54^ Assessment of dyspnoea is challenging because patient activity level affects their sense and the impact of dyspnoea on QoL. There is no agreed questionnaire for dyspnoea in CHF,^51^ so we have summarized the available tools for the assessment of dyspnoea (*Tables* *1* and *2*). While many of these questionnaires have been validated for reproducibility with some correlating to prognosis, in general, they lack sensitivity to acute changes in dyspnoea or specificity to CHF with some too long or under copyright to be practical for routine clinical use.^51^Treating dyspnoea requires assessment of the cause, which in people without pulmonary oedema remains controversial. If due to pulmonary or peripheral congestion, loop diuretics are highly effective with renin–angiotensin system antagonists useful in preventing reaccumulation. Device therapies such as cardiac resynchronization therapy and left ventricular assist devices are also associated with reduced dyspnoea.^58^, ^59^ A greater focus on symptoms could enable promising alternative therapies such as relaxin and sildenafil to explore further which have been yet to be formally approved due to unimproved mortality and hospitalization.^60^

**Table 2:** Summary of dyspnoea‐focused questionnaires not yet validated for CHF

Name of assessment tool	Description	Structure	Strengths	Limitations
Borg Scale	Assesses dyspnoea during cardiopulmonary exercise testing	● Dyspnoea during exercise is ranked 0–10 (0 = no perceived dyspnoea and 10 = maximal dyspnoea)	● Simple to use and commonly utilized in the research setting	● It is estimated one in every two to three CHF patients are unable to conduct CPET appropriately.^55^, ^56^
Chronic Respiratory Disease Questionnaire	Assesses impact of dyspnoea on overall well‐being; similar to the Chronic Heart Failure Questionnaire	● Dyspnoea is rated using 1–7 scale, where 1 = extremely breathless and 7 = not breathless at all, in relation to five activities of daily living (ADLs) selected by patient.	● Patient‐specific survey	● Patient specificity makes this tool less useful for inter‐patient comparisons.
		● Includes standardized questions regarding emotional function and fatigue		
Medical Research Council Dyspnoea Scale	Assesses dyspnoea in relation to ADL	● Patients give a 1–5 score, ranging from 1 being ‘not troubled by breathlessness except on strenuous exercise’ to 5 being ‘too breathless to leave the house, or breathless when undressing’.	● Can be used in follow‐up visits to track change in dyspnoea	● Lacks sensitivity to track responses to therapy in a single hospital stay, therefore inappropriate for hospitalized patients
	Designed for COPD patients			● This method has not been validated specifically in relation to CHF.
Oxygen cost diagram	Assesses dyspnoea in relation to ADLs	● Rating corresponding to the oxygen requirements of 13 different activities ranked from 0 to 100	● Indicates patient's perception of their exercise tolerance	● Subjective—does not correlate well with objective changes to exercise capacity
		● Sitting, sleeping, or standing are ranked close to 0 as they are low oxygen demand. Walking briskly/uphill would be closer to 100.		
		● A score of 100 indicates no impairment		● CHF patients may be incapable of completing all 13 ADLs, due to co‐morbidities or other symptoms, thus reducing value of this approach.
St. Georges Respiratory Questionnaire	Assesses impact of dyspnoea on overall well‐being	● Self‐completed form of 76 questions measuring symptom frequency and severity (rated with a 0–5 Likert scale) and their relation to ADLs (yes/no questions)	● Comprehensive	● Lengthy
	Designed for respiratory patients	● Question sections are weighted and scored to produce a cumulative 0–100 score, where a higher score indicates higher symptom impact.	● Associated with prognosis in selected patient cohorts such as idiopathic pulmonary fibrosis^57^	● This method has not been validated specifically in relation to CHF.
University of California San Diego Shortness of Breath Questionnaire	Assesses dyspnoea in relation to ADL	● Patients answer questions on a scale of 0 (no breathlessness) to 5 (unable to complete due to breathlessness).	● Comprehensive	● Lacks sensitivity to track changes across a day or week
		● Consists of 21 questions about the severity of dyspnoea associated with various ADLs		● CHF patients may be incapable of completing some ADLs in questionnaire due to co‐morbidities or other symptoms, thus reducing value of this approach.
		● Additional three questions focus on physical activity limited by dyspnoea or the fear of dyspnoea on the average day. This gives an overall score of 0–120.		

CHF, chronic heart failure; COPD, chronic obstructive pulmonary disease; CPET, cardiopulmonary exercise testing.

All instruments included have been tested for reliability.^51^

## Cough

Cough is a protective reflex that encourages the clearing of secretions or foreign particles from the larynx, trachea, and large bronchi. The mechanism is triggered by irritation of mechanical and chemical receptors located in trachea, bronchi, and smaller airways.^61^ Despite over 40% of CHF patients complaining of cough, it remains a symptom that is missed from commonly used tools such as the NYHA scale.^3^, ^11^ In CHF, cough is commonly either due to pulmonary congestion or secondary to angiotensin‐converting enzyme inhibitors through an accumulation of bradykinin and prostaglandins.^62^Persistent cough causes breathlessness, fatigue, and chest pain, disrupting activity and sleep.^1^, ^18^, ^63^ Chronic cough can also have a considerable psychological impact on CHF patients through inconvenience, embarrassment, frustration, and incontinence, thereby contributing to depression.^64^, ^65^ On the other hand, an inability to efficiently cough, for instance, due to fatigue or breathlessness, can cause increased susceptibility to infection due to secretion retention.^66^Cough is often overlooked in clinic, even by the patients and carers themselves in favour of issues such as dyspnoea and fatigue. Hence, although cough is highly prevalent in CHF, there is little literature addressing its impact and formal assessment. Assessment of cough involves exploring several components including intensity, frequency, and disruptiveness.^67^ *Table* *3* provides a summary of three cough assessment tools, although none is especially frequently used. These symptom surveys could be used in conjunction with ambulatory cough monitors to assess cough frequency, severity, and impact on QoL.^64^, ^66^, ^68^ While these surveys have been shown to be well validated and responsive, none have been designed or validated for CHF specifically. Cough is also included in some wider symptom surveys, such as the Memorial Symptom Assessment Scale and the Symptom Distress Scale (*Table* *4*).The treatment of cough in CHF is incomplete and focuses around reducing pulmonary oedema with the use of diuretics and removing possible contributors such as angiotensin‐converting enzyme inhibitors. There is a need to develop further management options.

**Table 3:** Summary of cough focused questionnaires not yet validated for CHF

Name of assessment tool	Description	Structure	Strengths	Limitations
Chronic Cough Impact Questionnaire	Assesses global impact of cough in relation to QoL	● 21‐item self‐administered questionnaire covering four health domains: daily activities, social relationships, mood, and sleep/concentration	● Well validated, repeatable, and responsive	● This tool has not been validated specifically in relation to CHF.
Cough‐specific Quality of Life Questionnaire	Assesses global impact of cough in relation to QoL	● 28‐item self‐administered questionnaire covering six health domains (physical complaints, extreme physical complaints, psychosocial issues, emotional well‐being, personal safety fears, and functional abilities)	● Well validated, repeatable, and responsive	● This tool has not been validated specifically in relation to CHF.
			● Validated in other languages	
			● Can be used to assess health status in acute cough	
Leicester Cough Questionnaire	Assesses global impact of cough in relation to QoL	● 19‐item self‐administered questionnaire covering three health domains (physical, psychological, and social) are scored using a 7‐point Likert scale.	● Well validated in clinical and research setting	● This tool has not been validated specifically in relation to CHF.
			● Concise and self‐administered	

CHF, chronic heart failure; QoL, quality of life.

All instruments included have been tested for reliability.^66^, ^68^

**Table 4:** Evaluation of existing PRO assessment tools that could be utilized in CHF

Name of assessment tool with validation reference	Structure	Strengths	Limitations	Validated in CHF
Cardiac Health Profile of Congestive Heart Failure	● 10‐item self‐assessment tool	● Correlates well with the MLHFQ, maximal workload during exercise and NYHA	● Women were poorly represented in the validation study.	Yes^69^ ^,^ ^121^
	● Covers both disease‐specific and general areas of heart failure issues			
Care‐Related Quality of Life survey for Chronic Heart Failure	● 20‐item self‐assessment tool covering a range of concerns from physical, emotional, and social	● Adds new concepts to CHF assessment primarily around patient anxiety	● Requires further validation work into the discriminatory properties of the tool	No
Chronic Heart Failure Questionnaire	● 16‐item interview‐administered assessment tool	● Able to detect changes over time	● Interviewer required	No
			● Can be difficult to conduct inter‐patient analysis	
	● Mainly focuses on dyspnoea, fatigue, and emotional impact	● Personalized to the patient	● Requires licensing for use	
Edmonton Symptom Assessment Scale	● 10‐item self‐assessment tool	● Can be self‐administered	● Not heart failure specific	Yes (including revised version)^70^
	● Uses 0–10 to rate their level of distress from pain, fatigue, nausea, depression, anxiety, sleepiness, appetite, dyspnoea, and ‘other’ symptoms	● Widely used		
	● Initially developed for cancer patients	● Actively developed for further utilization		
Heart Failure Somatic Awareness Scale	● 12‐item self‐assessment tool to measure awareness of and distress secondary symptoms	● Simple to use and relatively quick to use	● Small population for validation study	Yes^71^
Kansas City Cardiomyopathy Questionnaire	● 23‐item self‐assessment tool covering six domains: physical limitation, symptom, symptom stability, self‐efficacy, QoL, and social limitation	● Can be self‐administered	● Lengthy	Yes (including short version)^72^
		● Can be completed within 10 min		
		● Sensitive to changes in symptoms	● Requires licensing for use	
The Left Ventricular Dysfunction Questionnaire	● 36‐item self‐assessment tool, which are answered true or false	● Useful for monitoring change in symptoms and scores correlated well to the patient's perception of change	● Small population for validation study with low representation of women	Yes^73^
	● The answers are aggregated to produce eight component scores and two overall summary			
	● Scores which run from 0 (worst) to 100 (best score)			
Multidimensional Index of Life Quality	● 35‐item self‐assessment tool covering nine domains ranging from physical health and function to financial status and social circumstances	● Depicts global QoL	● Not heart failure specific	No
			● Some domains score poorly on retest reliability.	
MLHFQ	● 21‐item self‐assessment tool covering three domains: physical, socioeconomic, and psychological	● Can be self‐administered	● Lengthy	Yes^74^
		● Can be completed within 10 min		
	● Mainly focuses on dyspnoea, fatigue, and emotional aspects	● Widely used	● Requires licensing for use	
Memorial Symptom Assessment Scale‐Heart Failure	● 32‐item symptom assessment scale	● Comprehensive and well validated	● Lengthy	Yes^11^
	● Three symptom subscales: physical, emotional, and heart failure‐specific symptoms			
	● Uses Likert scales to rate overall frequency, intensity, and distress associated with 35 common symptoms			
Symptom distress scale	● 15‐item assessment tool	● Personalized to the patient	● Not heart failure specific	No
	● Uses Likert scales to assess impact of symptoms		● Ambiguity in interpretation of questions	
	● Mainly focuses on fatigue, insomnia, mood, mobility, concentration, breathing, pain, nausea, and appearance			
Short Form Health Survey	● 36‐item self‐assessment tool covering two domains: physical health and mental health	● Can be self‐administered	● Not heart failure specific	Yes^75^
	● Uses Likert scales to assess pain, general health, vitality, social functioning, emotional, and mental health	● Depicts global QoL		
Sickness impact profile	● 68‐item generic health measure	● Depicts global QoL	● Not heart failure specific	No
	● Assesses autonomy, mobility, behaviour, feelings, and communication		● Lengthy	
Symptom Status Questionnaire–Heart Failure	● 7‐item self‐assessment tool for measuring the patient's perception to physical CHF symptoms with five response options ranging from 0 (none) to 4 (experienced nearly daily)	● Short and easy to complete	● Narrow focus of CHF symptoms experienced	Yes^76^
Quality of Life Questionnaire in Severe Heart Failure	● 26‐item self‐assessment tool with a combination of visual analogue and Likert scales	● Includes emotional and cognitive aspects of QoL in addition to general satisfaction	● Small population for validation study	Yes^77^
			● Limited utilization in modern CHF research	

CHF, chronic heart failure; MHLQ, Minnesota Living with Heart Failure Questionnaire; NYHA, New York Heart Association; QoL, quality of life.

## Peripheral oedema

Peripheral oedema, found in over 50% of CHF patients, is a well‐known sign of the condition but also features as a symptom.^50^ It is normally a result of right‐sided heart congestion and can range from mild episodic ankle swelling to severe generalized fluid retention.^78^ While oedema commonly manifests in the distal limbs, severe peripheral oedema can present alongside ascites, scrotal congestion, and even subconjunctival oedema.^78^ Oedema is uncomfortable at best and can limit exercise capacity, resulting in disturbed sleep, pain, and increased risk of infection. Early detection of oedema may avoid complications such as ulcers, bed sores, stasis eczema, and cellulitis. Unfortunately, patients often fail to appreciate mild peripheral oedema due to its insidious development and mistake it for normal weight gain^63^, ^78^ such that oedema may not be reported until 20 L of fluid has accumulated. Many patients have little appreciation of the significance or the knowledge of how to adjust their diuretic dose.^63^, ^78^, ^79^ Education to facilitate self‐care is required to tackle this issue.^80^ Peripheral swelling is included in most symptom questionnaires because weight gain can be masked by cachexia.^63^ Newer optical scanners utilize non‐contact depth sensing methods to create advanced 3D images for determining changes in leg shape, size, and consistency.^81^ Hence, novel approaches to oedema monitoring are needed to build a model of disease progression and facilitate patient‐directed diuretic dose adjustment, balancing the risk of renal impairment.Pharmacological treatment for peripheral oedema is focused around diuretics and mineralocorticoid receptor antagonists with further medications to prevent formation through disease management. Exercise and investigating drug interactions such as the stoppage of dihydropyridine calcium channel blockers are also of value.^78^ The role of interventions such as ultrafiltration in treatment‐resistant peripheral oedema remains unclear and requires further study with potentially greater emphasis placed on changes to QoL and symptom burden.^82^

## Pain

Pain is an under‐recognized yet debilitating symptom of CHF, which can range in characteristic from musculoskeletal ache, deep visceral pain, and neuropathic pain, all of which are reported at a similar prevalence to dyspnoea in end‐stage CHF.^10^, ^50^, ^83^, ^84^ For example, the PAIN‐HF study reported that 84% of 347 patients with advanced CHF complained of pain, and 70% believe it interfered with activities of daily living. While pain was most commonly cited in the legs and back, more than a third experience pain in multiple sites.^85^ Pain can be challenging to classify and find the source,^86^ particularly in CHF where ageing, co‐morbidities, and general deconditioning commonly coexist.^50^ Pain also has significant overlap with other CHF symptoms such as breathlessness, low mood, and poor sleep. One overlooked adverse effect of pain is its autonomic response, which can further activate the renin–angiotensin–aldosterone cascade.^87^ Pain severity is usually rated on a scale of 1–10. Its subjective nature is problematic, particularly for inter‐patient comparisons. A carefully taken history coupled with standardized and repeated assessment has a key role. Lower levels of pain or good pain control are associated with better medication adherence, improved ability to self‐report symptoms and self‐care implying benefits beyond simply improved QoL.^87^, ^88^ For example, the diagnosis of chronic pain makes patients four times more likely to be diagnosed with depression.^89^ The most common treatments for pain include paracetamol, non‐steroidal anti‐inflammatory drugs, and opiates. Opiates have an important role in CHF both in the early and later palliative stages of the disease and are safe, well tolerated, and effective.^85^ Conversely, non‐steroidal anti‐inflammatory drugs can increase the risk of progression of CHF as well as the frequency of adverse events, making its use controversial.^90^ A number of non‐medical alternatives have been suggested, such as the use of hot or cold patches and stretching exercises.^91^ As with the other less frequently discussed symptoms, there are limited data available on appropriate analgesia or non‐medical intervention for pain relief in CHF. Moving to symptom‐focused pathways of care with repeated testing will help identify prevalence and course of symptoms and develop standardized approaches to the treatment of this distressing symptom.

## Low mood

A 2006 meta‐analysis of 36 studies determined that ~22% of CHF patients are diagnosed with ‘clinically significant’ depression.^92^ CHF‐associated depression is more common in patients with co‐morbidities, rapid disease progression, or younger age at presentation.^93^ Depression is also commonly overlooked due to overlapping signs and symptoms such as fatigue.^10^ Furthermore, the wide array of possible tools and thresholds of depressive symptoms often without standardization makes low mood difficult to assess confidently.The exact aetiology of low mood is often unclear in individual patients. CHF patients are at risk of feelings of worthlessness and guilt as they become increasingly dependent on carers.^94^, ^95^ This is often preceded by or can lead to a vicious cycle of reduced activity and motivation, worsening health status, and increasing dependence.^14^ Faris *et al*.^96^ studied 396 patients with CHF and found that in comparison with those without depression, depression was associated with worse symptoms of longer duration, higher risk of hospital admission, and a doubling of mortality rate. Once it is recognized, treatment of depression is associated with improved QoL and medical adherence.^97^ Moreover, because depression has an adverse effect on the autonomic nervous system, it could worsen the pathophysiological drivers of the syndrome,^96^ thereby explaining the heightened risk of disease progression and poorer overall outcomes including hospitalization rates and sudden cardiac death.^97^, ^98^, ^99^, ^100^, ^101^, ^102^ Worsening depression over a year is also a bad omen, hinting that regular monitoring could allow early intervention with the aim of improving outcomes.^100^A number of mood assessment tools are available including the Hospital Anxiety and Depression Scale, Geriatric Depression Scale–Short Form, and the Patient Health Questionnaire‐9 (*Table* *1*). These questionnaires are used variably across clinical environments and have a number of advantages and shortcomings (*Table* *3*).^90^ Only the Patient Health Questionnaire‐9 has been shown to correlate with QoL and readmission in CHF.^103^, ^104^Treating low mood can be done safely with pharmacological treatments such as selective serotonin reuptake inhibitors,^105^ whereas tricyclic antidepressants and monoamine oxidase inhibitors should be avoided because of the increased risk of arrhythmias or hypotension.^106^ Non‐pharmacological interventions such as psychotherapy, such as cognitive behaviour therapy and exercise, have also shown to be successful methods of managing depression in CHF but have limited availability.^107^, ^108^ Early identification and management of low mood may slow deterioration and may therefore improve CHF‐specific outcomes.

## Existing strategies for symptom assessment in chronic heart failure

Patient‐reported outcomes (PROs) are question‐based tools that quantify QoL by assessing symptom frequency and severity according to the patient's perspective that can be applied systematically at each encounter.^109^ Disease‐specific PROs can be used to form a picture of the patient's current disease status and overall well‐being both at baseline and compared with previous assessments. This enables a systematic approach for obtaining QoL data that are inexpensive and effective.^52^ PROs have been shown to provide a more accurate overall picture of disease status than physiological assessments such as left ventricular ejection fraction.^95^ Furthermore, a general clinical assessment of symptoms often varies by healthcare professionals such that structured questionnaires could improve consistency in clinical care.^110^ Despite a plethora of available tools (31 that we could find) with some use in clinical research, they are infrequently used to guide clinical practice.^95^ We have reviewed a number of PROs available (*Table* *4*) including the three most commonly cited questionnaires: the MLHFQ, the Kansas City Cardiomyopathy Questionnaire (KCCQ), and the Edmonton Symptom Assessment System–Revised. Developed in 1987, the MLHFQ provides scores based on physical and emotional symptoms. It is frequently used in CHF due to its ease and familiarity,^111^ and it predicts event‐free survival following CHF decompensation with utility in identifying changes in the patient's QoL and outcomes.^112^ The KCCQ quantifies health status with a higher score indicating better health predictive of hospitalization and cardiovascular risk.^111^ The KCCQ has since been shortened to consist of 12 questions, to improve accessibility.^113^, ^114^ This shorter questionnaire has positive correlations with the original, high test–retest reliability and responsiveness.^113^ Bekelman *et al*.^6^ concluded that KCCQ should be used as a clinical indicator for palliative needs; however, neither the KCCQ nor the MLHFQ comprehensively assesses physical, psychological, and social health contributors to QoL.^95^The Edmonton Symptom Assessment System has been validated and translated in over 20 different languages.^115^ It was originally created to document symptoms in end‐stage cancer patients requiring palliative care and has since been revised into a simpler and more patient‐friendly tool known as the Edmonton Symptom Assessment System–Revised. It is quick to complete and in contrast to other tools is highly accessible through generous licensing agreements.^84^Patient‐reported outcomes in CHF are validated and reproducible and are increasingly utilized as secondary outcomes in clinical trials such as the Angiotensin–Neprilysin Inhibition versus Enalapril in Heart Failure (PARADIGM‐HF) study^116^ and the upcoming Empagliflozin in Heart Failure Patients With Reduced Ejection Fraction (Empire HF) trial.^117^ In due course, given that death and hospitalization have a significant impact on patient QoL, it is feasible that PROs could move towards becoming primary outcome measures. Moreover, in patients reaching end of life, PROs could direct medical care away from measures to enhance survival and towards those that enhance remaining life.^118^ The utility of these tools may be enhanced further by being accessible for patients to help them assess their own health status.Outstanding issues around existing PROs that may explain the relative lack of uptake include cost, copyright, practical implementation, lack of breadth of symptoms, challenges around presentation, issues around credibility of results, and the focus on survival for approval of new interventions.^119^ Many of these issues would be solved with familiarity and validated modification for local needs. It is already the case that over 70% of healthcare professionals questioned by Wohlfahrt *et al*.^120^ believed that PRO assessment should become routine in clinical care, and emerging data confirm that integration into a standard CHF clinic is feasible and acceptable to patients. The restructuring of clinical services away from face‐to‐face reviews with a greater emphasis on digital technologies presents a challenge but one in which PROs could take a leading role for routine care and research activity. Indeed, this approach has been shown to be both feasible and valuable by Stehlik *et al*.^121^ in the clinic setting, finding the average time of completion to be 6.7 min with 91% of started assessments completed fully. We believe this approach will not only improve recognition of the plethora of manageable symptoms associated with CHF but also aid standardization, initiation of required therapy, assessment of intervention response, and the clinical consultation itself.

## Summary

Chronic heart failure leads to symptoms in patients across a range of domains that are frequently poorly assessed. We believe there are opportunities to improve patient contact episodes to identify underlying problems and improve clinical management in a holistic fashion. It seems both feasible and essential for a PRO to be implemented in a clinical environment such as outpatient clinics, thus adding value to the consultation and monitoring improvements in patients and the CHF population.

## Conflict of interest

A.K. is enrolled on a PhD fellowship programme partly funded through a collaborative unconditional research grant to the University of Leeds from Medtronic UK. K.K.W., M.T.K, and R.M.C. have received unconditional research support from Medtronic; K.K.W. has served as an advisor for Medtronic, an advisor, speaker, and proctor for Cardiac Dimensions, and a speaker and advisor for Novartis, Bayer, Napp, Pfizer, and Daiichi Sankyo. The authors have no other relevant affiliations or financial involvement with any organization or entity with a financial interest in or financial conflict with the subject matter or materials discussed in the manuscript apart from those disclosed.
